# Physical accessibility of primary care facilities for people with disabilities: a cross-sectional survey in 31 countries

**DOI:** 10.1186/s12913-021-06120-0

**Published:** 2021-02-01

**Authors:** Peter P. Groenewegen, Madelon Kroneman, Peter Spreeuwenberg

**Affiliations:** 1 NIVEL (Netherlands Institute for Health Services Research), PO box 1568, 3500BN Utrecht, The Netherlands; 2 Department of Sociology and Department of Human Geography, Utrecht University, P.O. Box 80.115, 3508 TC Utrecht, The Netherlands

**Keywords:** Physical accessibility, Disabled people, Primary care, Policy, International comparison

## Abstract

**Background:**

Primary care is the first point of care, also for people with disabilities. The accessibility of primary care facilities is therefore very important. In this study we analysed comparative data on physical accessibility of general practices (GP practices) in 31 (mainly) European countries.

**Methods:**

We used data from the QUALICOPC study, conducted in 2011 among GPs in 34 (mainly European) countries and constructed a physical accessibility scale. We applied multilevel analysis to assess the differences between and within countries and to test hypotheses, related to characteristics of the practices and of the countries.

**Results:**

We found large differences between countries and a strong clustering of physical accessibility within countries. Physical accessibility was negatively related to the age of the GPs, and was less in single-handed and in inner city practices. Of the country variables only the length of the period of social democratic government participation during the previous decades was positively related to physical accessibility.

**Conclusion:**

A large share of the variation in physical accessibility of GP practices was on the level of countries. This means that national policies can be used to increase physical accessibility of GP practices.

**Supplementary Information:**

The online version contains supplementary material available at 10.1186/s12913-021-06120-0.

## Background

Accessibility of primary care facilities is increasingly important with more people with a disability living in the community, partly as a result of ageing of the population [1]. However, the trend in deinstitutionalisation of people with disabilities is not the same everywhere and there are both countries with a decreasing and with an increasing number of people with disabilities who live in institutions [2]. Accessibility of primary care includes several dimensions, such as geographical accessibility, affordability, accessibility of the accommodation, and timeliness and acceptability of services provided [3]. In the context of people with disabilities, our focus is on physical accessibility of the practice accommodation.

A report from the European Union Agency for Fundamental Rights shows large variation between EU member states in access to general services for people with disabilities living in the community, with a range of 25 to 65% declaring difficulty in accessing at least one of five types of services (including banks, public transport and primary health care services). Among persons with disabilities, on average 23% declared to have difficulties in accessing primary health care and this represents the biggest gap between people with and without disabilities among these general services [4] (p. 86). Primary care is of particular importance, because it has a coordinating role between different health, care and support services for people with disabilities [1]. Lack of access to health care has consequences for health and wellbeing of people with disabilities [5] and leads to unmet health needs [6, 7].

There are different approaches in the literature on access to (primary) care for people with disabilities: starting from the experiences of people with a disability seeking (primary) health care [8, 9], from the views of care providers [8] and from assessments of (primary) health care facilities in terms of their accessibility, often in relation to norms for good access [10–12]. However, much of the literature are studies in the USA [6] and there is a lack of international comparisons. The World Disability Report provides information either on selected countries or on a high level of aggregation: high versus low income countries [1].

In this article we focus on the physical accessibility of primary care facilities; consequently, this relates mainly to people with mobility disabilities. However, physical accessibility is more generally relevant for older persons and persons accompanying babies and toddlers. We fill in the gap in knowledge by providing an international perspective. The data we will use were collected between 2010 and 2012; it is, however, the only data of this scale, as far as we know.

International differences in how accessible primary care facilities are for persons with a disability may be related to direct policies in this area. Governments may have implemented policies relating to the accessibility of health care facilities. There may also be more general policies on access to community services and public buildings for disabled people. Finally, there may be policies in other areas, such as disability benefits, access to public transport, or active labour market policies to include people with a disability that indicate a general disposition towards disability inclusive policies. This general policy disposition may be related to the mainstream political ideas and welfare regime in a country [13]. When liberal laissez-fair politics are prevailing, there may be less political support for supportive or compensating policies for people with a disability. Conservative/corporatist politics tend to leave policies for people with a disability to the ‘social partners’, the employers and employees, with few possibilities for government to set standards or to push policy changes. Social democratic politics might put more emphasis on equity in different realms of life, including policies for people with a disability. That politics matters in health and welfare policies, is shown by the international literature [14]. Policies for the disabled in the health field may also be affected by the introduction of market elements in health care and austerity measures [15]. If there are policies that directly relate to accessibility of (primary) health care facilities, these are easier to implement in centralised and national health systems than in decentralised systems or systems with private healthcare providers [16]. Finally, differences between countries could also be related to their level of wealth. In richer countries there will be more room to invest in adaptations of public buildings and transport in general and perhaps also of health care facilities.

Primary care practices may differ in physical accessibility within countries. This may be related to the age of the premises with newer buildings being better accessible in general [12], and the size of the premises with bigger buildings perhaps being better accessible. Also the place where the primary care practice is located may be of influence. We expect that practices in rural areas pose more problems of physical access for people with disabilities [17]. This may be related to the age and size of the premises but also to a lower sensitivity to problems of people with a disability in rural compared to urban areas [18]. The study by Mudrick et al. did not find the expected urban-rural difference and they suggest that this may be explained by newer premises in the non-urban settings in their sample [11]. In the same line of reasoning we may expect that primary care facilities in deprived areas pose more access barriers to people with disabilities, although we are not able to back this up from the literature.

In this article we will explore variation in physical accessibility of primary care facilities for people with disabilities in 31 countries. Our first question is to what extent accessibility of primary care practices varies between countries and between practices.

Secondly, we would want to know what it is in countries or in practices that relate to accessibility of the facility for people with disabilities. The previous discussions related to country and practice level influences on physical accessibility of primary care practices can be summarised in the following hypotheses:

- Primary care practices will be better accessible in countries that have implemented national policies on accessibility of general services for people with disabilities, in countries with a longer period of social-democratic government participation, in countries with a more centralised healthcare system, and in wealthier countries.
- Primary care practices will be better accessible if they are more recently built, if they are bigger, if they are located in less rural areas and in less deprived areas.

## Methods

### Data and measurements

We used cross-sectional data collected in the Qualicopc (Quality and costs of primary care in Europe) study between 2010 and 2012 [19]. For this study, primary care practices were sampled in 34 (mainly European) countries [20]. Around 220 general practitioners (GPs) per country participated, except for the smaller countries (Cyprus, Iceland, Luxembourg, and Malta) where this was around 75 GPs. For the UK, only GP practices in England were sampled. In Canada, Belgium, and Spain, larger samples were taken to represent different regions. In most countries, a random sample was invited to participate. Where no national sampling frame was available, alternatives were sought as close as possible to a random sample. Per practice, only one GP participated. The response among GPs was on average 30% and the response group mirrored the national GP populations in terms of age and gender.

Data collection among patients of these practices was done by field workers who also filled out a brief questionnaire about the practice. Denmark and New Zealand were left out, because no field workers were used; Portugal had missing values on one of the independent variables, leaving us with 31 countries for statistical analysis. In the practice questionnaire the following questions about physical accessibility were asked:

- The practice has parking space for disabled people (Yes/no)
- Is the practice on the ground floor? (Yes/no)
- If not on the ground floor: Is an elevator available for patients? (Yes/no)
- How accessible is the practice for patients using a wheelchair or stroller? (very easy, easy, difficult, impossible to access)
- Is a toilet available for patients with a disability? (Yes/no)

The questionnaires used in the QUALICOPC study were developed on the basis of existing, validated questionnaires [21]. The questions on physical accessibility are inspired by and partly based on Engels et al. [22].

We used the GP questionnaire to measure potential correlates of physical accessibility at the level of the practice and the task environment of the practice:

- shared practice two or more GPs (compared to single-handed); we used this variable because a direct measure of the size of the practice building was not available in the data;
- age of the GP; our assumption is that on average older GPs will be working in older premises;
- the location of the practice (four categories of urbanisation);
- the composition of the practice population in terms social deprivation (above average, average or below average of the country).

Information on country level variables was collected from external sources. Information on national policies concern the date of ratification of the UN Convention on the Rights of Disabled People and on the regulation of accessibility of buildings. Source for the latter is the Country Reports on Human Rights Practices for 2012 from the US Department of State, section 6 Discrimination, societal abuses, and trafficking in persons; persons with disabilities [23]. We have built an ‘accessibility regulation index’ using the information in this report. This information reflects the situation around the period that the data on accessibility of GPs was collected. To create the index we used the following steps:

- Countries that have laws or regulations in place requiring accessibility for buildings score three points;
- When the regulation is explicitly restricted to public buildings, one point is subtracted;
- When enforcement or implementation is considered problematic as reported in the Country Reports on Human Rights Practices, one point is subtracted. When no information is provided on enforcement or implementation, it is considered to be reasonable successful;
- When no such regulation exists, a country scores zero points.

Supplementary Table 1 contains the information used and the resulting score for each country.

The general political will to equalise the life chances for all inhabitants irrespective of disabilities was operationalised by the number of years the social-democratic parties participated in government between 1993 and 2010, weighted for their share in government [24]. This information is at the level of countries; however, it may be argued that the more appropriate level in federal sates would be regions or states within the federation. We therefore also conducted a sensitivity analysis, excluding federal states (in this case: Australia, Austria, Belgium, Canada, Germany, Italy, Spain and Switzerland, following [25]). Health care systems were classified as either centralised or decentralised, measured as the responsibility for the distribution of money in the health care system. Countries where this responsibility lies with central government were classified as centralised; countries where this responsibility lies with multiple parties, such as insurance companies or local regions, were classified as decentralised (sources: Various health care systems in Transition Profiles of the European Observatory on Health Systems and Policies).

To indicate the affordability of measures to make buildings accessible we have used Gross Domestic Product (GDP) per head of the population in ‘PPP constant 2011 international $’ for 2012 (source: Worldbank [26]).

### Statistical analysis

The physical accessibility of GP practices was described using the five items from the practice questionnaire. The analysis was done using multilevel analysis to take the nested structure of the data into account. The physical accessibility scale was constructed in an ecometric model with the items nested within GP practices and practices in their turn nested within countries [27]. The item whether or not an elevator was available, was only included in the analysis if the practice was not on the ground floor [28]. The ecometric approach also makes it possible to calculate the reliability (both at practice level and at country level). We used the formula developed by Raudenbush, which gives reliability comparable to Cronbach’s alpha in single level analysis [29].

We used the random effects (variances) at GP practice and country level to describe the clustering of physical accessibility. The country level variances were used to construct a caterpillar plot to show the differences between countries on the physical accessibility scale. The GP practice and country variables were entered in a multilevel linear regression analysis with the scale value as dependent variable. A higher scale value means better accessibility of practices. Age of the GPs, size of the practice and social deprivation of the practice population were entered as continuous variables. Practice location was entered as a set of dummy variables with (inner) city as the reference category. For the GP and practice characteristics we used listwise deletion of missing values. As the number of countries is relatively small for statistical analysis, we included these variables one at a time. For the same reason we use *p* < 0.10 as the boundary value for statistical significance for the country level variables and the conventional *p* < 0.05 for the GP practice/location variables.

Analyses were performed in MLwiN, version 2.30.

### Ethical approval

Ethical approval for the QUALICOPC study was acquired in accordance with the legal requirements in each country [30].

## Results

### Description of physical accessibility

Descriptive information on the accessibility of practices was available for 6566 practices in 32 countries. The distribution of the five items over the countries is given in Table 1.

**Table 1:** Physical accessibility of GP practices in 32 countries; percentage yes and missing values per item

Country	Parking space for disabled people; % yes (n missing)	Practice on the ground floor % yes (n missing)	If not on ground floor, elevator % yes (n missing)	Wheelchair or stroller accessible % very easy (n missing)	Toilet for patients with disability % yes (n missing)	N
Australia	73 (4)	88 (1)	69 (3)	60 (1)	70 (3)	130
Austria	17 (2)	51 (0)	61 (0)	25 (2)	22 (6)	180
Belgium	27 (4)	89 (3)	13 (4)	23 (5)	15 (8)	407
Bulgaria	49 (3)	64 (5)	47 (2)	24 (1)	27 (3)	222
Canada	92 (1)	65 (0)	92 (0)	60 (0)	91 (1)	515
Cyprus	63 (0)	92 (2)	100 (2)	20 (0)	44 (3)	71
Czech Republic	49 (7)	55 (0)	72 (0)	32 (1)	57 (1)	220
England	89 (1)	57 (1)	65 (2)	56 (0)	97 (0)	159
Estonia	44 (3)	54 (0)	66 (0)	33 (53)	48 (3)	125
Finland	87 (5)	70 (0)	90 (2)	61 (0)	83 (3)	138
FYR Macedonia	39 (5)	59 (1)	17 (0)	20 (0)	12 (0)	143
Germany	38 (4)	51 (2)	54 (1)	32 (0)	23 (4)	236
Greece	31 (7)	96 (1)	100 (2)	63 (1)	47 (4)	220
Hungary	48 (6)	88 (1)	54 (0)	37 (1)	53 (3)	220
Iceland	91 (0)	51 (3)	98 (2)	68 (0)	93 (5)	87
Ireland	77 (2)	78 (5)	62 (0)	64 (2)	87 (3)	192
Italy	26 (6)	66 (2)	79 (2)	31 (2)	36 (6)	218
Latvia	50 (4)	58 (2)	64 (3)	28 (1)	52 (0)	218
Lithuania	72 (0)	52 (3)	84 (5)	25 (0)	48 (8)	225
Luxembourg	38 (2)	61 (0)	67 (1)	22 (1)	26 (3)	80
Malta	46 (0)	46 (0)	79 (0)	18 (2)	39 (3)	70
Netherlands	50 (1)	90 (0)	91 (0)	36 (1)	54 (10)	227
Norway	76 (7)	56 (0)	100 (1)	50 (1)	89 (2)	204
Poland	46 (1)	80 (0)	74 (0)	37 (0)	84 (0)	219
Portugal	79 (3)	61 (3)	93 (0)	43 (0)	85 (5)	216
Romania	37 (1)	81 (0)	10 (0)	26 (1)	19 (0)	220
Slovakia	47 (1)	30 (1)	90 (2)	11 (2)	61 (5)	220
Slovenia	88 (2)	47 (0)	87 (1)	40 (0)	53 (4)	220
Spain	78 (7)	72 (0)	81 (3)	48 (1)	96 (2)	215
Sweden	98 (1)	49 (0)	95 (2)	93 (0)	100 (0)	43
Switzerland	46 (6)	42 (0)	84 (4)	42 (0)	46 (4)	200
Turkey	35 (5)	77 (1)	23 (1)	30 (0)	44 (2)	293

The overall mean of the physical accessibility scale was 0.604 (median 0.625; 5th percentile 0.303 and 95th percentile 0.865). The reliability of the scale at practice level was 0.62; at country level it was 0.99.

**Fig. 1:**
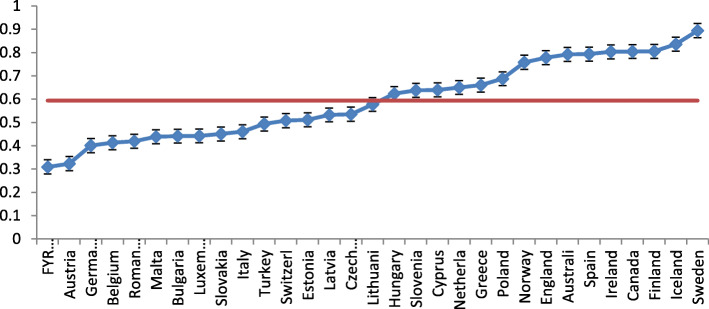
Caterpillar plot of physical accessibility of GP practices in 31 countries, country score and confidence intervals and average score (red line,) based on the empty model in a linear multilevel regression analysis

Figure 1 shows the scale values by country in a caterpillar plot, with their confidence intervals. This shows the large differences between countries in physical accessibility of GP practices. The highest accessibility is found in Canada, Finland, Iceland and Sweden; the lowest in the FYR Macedonia, Austria, Germany and Belgium.

### Clustering of accessibility within countries

We assumed that the physical accessibility of GP practices for disabled people is influenced by characteristics of the GP practices and their task environment as well as national policies. This should be visible in the degree to which physical accessibility clusters within practices and countries, as expressed in the intraclass correlation. In line with the country differences in Fig. 1, the ICC is very high; 78% of the variance is on the country level (see Table 3, random effects empty model). The ICC can be seen as a measure of the correlation between practices within countries.

### Correlates of accessibility

All countries in our sample have ratified the UN Convention on the Rights of Disabled People. However, they differ in how long ago they did so compared to the date of the data collection of the QUALICOPC study (2011). The first countries to ratify the UN Convention in our sample are Italy and Hungary and among the last to do so until 2011 were Estonia and Malta (see Table 2). The accessibility index is lowest in Estonia and high in (amongst others) Australia, Germany, Lithuania and Spain.

**Table 2:** Country level variables: years since ratification of the UN Convention, accessibility regulation index, and GDP per capita

Country	Years since ratification UN Convention^**a**^	Accessibility regulation index	Gross Domestic Product per head (PPP)	Years of social-democratic government^**b**^	Centralised health system (yes/no)
Australia	4	3	42,855	6.50	no
Austria	4	1	44,552	5.75	no
Belgium	3	1	40,990	8.25	no
Bulgaria	0	2	15,772	4.25	yes
Canada	2	2	41,845	0	no
Cyprus	1	1	31,750	4.50	yes
Czech Republic	3	2	28,527	6.25	no
England^c^	3	2	37,450	13.00	yes
Estonia	0	0	25,643	5.00	yes
Finland	−1	2	40,154	7.00	no
FYR Macedonia	1	1	11,548	No data	yes
Germany	3	3	42,640	9.00	no
Greece	0	2	24,364	11.75	no
Hungary	5	1	22,647	10.00	no
Iceland	−1	1	41,077	3.50	yes
Ireland	−1	3	44,766	3.25	yes
Italy	5	1	35,411	4.50	no
Latvia	2	2	20,865	5.25	yes
Lithuania	2	3	24,019	8.50	yes
Luxembourg	1	3	89,505	6.50	no
Malta	0	3	29,121	2.00	yes
Netherlands	-1	1	45,949	6.25	no
Norway	-1	2	62,923	10.25	no
Poland	0	2	23,218	7.00	no
Portugal	3	2	25,788	7.75	no
Romania	1	2	18,361	9.50	no
Slovakia	2	2	26,499	5.75	no
Slovenia	4	1	28,109	9.50	yes
Spain	5	3	30,905	10,00	no
Sweden	4	2	43,897	12.00	no
Switzerland	-1	2	56,150	4.50	no
Turkey	4	2	20,259	No data	no

^a^ Years since data collection; countries that ratified after 2012 have the value −1

^b^ Weighted years of social-democratic government participation 1993–2010

^c^ The first four variables apply to the UK; the variable ‘centralised health system’ applies to England only, as the GP practices were located in England

Of the practice and location variables, shared practices (as compared to practices with only one GP) are better accessible (see Table 3, fixed effects). Practices of older GPs (as a proxy for the age of the premises) are less accessible. Finally, (inner) city practices are less accessible than those located in less urban areas. The deprivation of the practice population is not related to physical accessibility. The explanatory power of the practice variables is minimal (the practice variation decreases by 1 % when comparing the empty model and the full model (with all practice variables).

**Table 3:** Linear multilevel regression analysis of physical accessibility of GP practices in 31 countries. Coefficient (standard error) (for empty model and model with practice/location variables: n_countries_ = 31; n_practices_ = 5865)

Fixed effects	Empty model	Model with practice/location variables
Constant	0.59 (0.030)	0.58 (0.029)
***Practice/location***		
Shared practice (yes = 1)		0.011 (0.003)**
Age of the GP		−0.0005 (0.0001)**
Urbanisation (ref. = inner city)		
Suburbs/towns		0.015 (0.003)**
Mixed urban-rural		0.018 (0.004)**
Rural		0.024 (0.004)**
Social deprivation of practice population		0.001 (0.002)
***Country*** ^***a***^		
Years since ratification UN Convention (n_countries_ = 31; n_practices_ = 5865)		−0.007 (0.014)
Accessibility regulation index (n_countries_ = 31; n_practices_ = 5865)		0.034 (0.036)
Centralised healthcare system (n_countries_ = 31; n_practices_ = 5865)		− 0.001 (0.061)
Gross Domestic Product per head (n_countries_ = 28; n_practices_ = 4922)		0.000 (0.000)
Years social democratic government (n_countries_ = 27; n_practices_ = 4655)		0.018 (0.010)*
**Random effects**		
Practice level variance	0.008 (0.0001)	0.008 (0.0001)
Country level variance	0.027 (0.007)	0.021 (0.0058) ^b^
ICC	0.779	0.728

* *p* < 0.10

** *p* < 0.01

^a^ Country level variables were included one at a time

^b^ In the model with Years social democratic government as independent variable at country level

Of the country level variables only the number of years of social democratic government participation is related to the physical accessibility of GP practices. The country level variation is reduced by 23%, when comparing the empty model and the model that includes the practice variables plus the variable that indicates social democratic government participation. The result of the sensitivity analysis, excluding eight federal states, do not differ much; the estimate of the regression coefficient is slightly smaller (0.018 in the model with all countries and 0.016 in the model excluding federal states), together with the smaller number of countries resulting in a slightly higher *p*-value (*p* = 0.071 in the original and *p* = 0.109 in the model excluding federal states).

## Discussion

Our study shows large differences in physical accessibility between the (mainly) European countries in our sample. There is also variation between GP practices within countries. Consequently, where people with a mobility handicap happen to live, influences whether they are able to easily access primary care facilities.

We formulated beforehand hypotheses that might explain variation within and between countries. Most of our hypotheses about variation between GP practices within countries were confirmed. Larger facilities (as indicated by whether or not more than one GP is practising at the facility) and newer facilities (as indicated by the age of the GP) are better physically accessible. Inner city practices appeared less accessible. Whether practices are located in deprived areas (as indicated by an above or below average share of socially deprived people in the practice population) is unrelated to physical accessibility. Countries with many single-handed practices (such as Austria and Belgium) are consequently at the lower end of the distribution of accessibility.

Of the country level hypotheses only one was confirmed. Countries with a longer period of social democratic government participation have better physically accessible GP practices. Our interpretation is that compensating policies for people with a disability will be more often supported by social democrats and that this support makes a difference for the accessibility of GP practices when they participate in government. It could be argued that social-democratic government participation is less relevant in federal states. However, also in unitary states, many aspects of welfare state policies are implemented at local or regional levels, whilst still influenced by politics at national level. The influence of national political participation may be less influential in federal states than in unitary states. However, this does not imply that there is no influence. The results of the sensitivity analysis showed similar results when excluding federal states. The review by Falkenbach et al. [14] cites research that shows that also in countries with divided powers, such as in federal states, healthcare spending is negatively influenced by right-wing government participation.

The more specific variables related to pro-disability policies (ratification of the UN Convention and our self-constructed Accessibility Regulation Index) were not related to physical accessibility of GP practices. The number of countries, in this case 31, is relatively small for hypothesis testing and it is impossible to evaluate the effects of separate variables in multi-variable analysis at country level (as is possible with the large numbers of observations at GP practice level). Looking at Fig. 1, there is a geographical component in physical accessibility – with countries from Central and Eastern Europe more often less accessible – which may be related to variables that are not covered by our country level variables and which may confound the hypothesised relationships.

The focus of our analysis was on physical accessibility for people with disabilities. This is a consequence of our use of existing data that were not collected to assess accessibility for people with disabilities in a broad sense. Physical access is only one aspect of accessibility of primary care facilities for people with disabilities [3]. Moreover, accessibility is broader than just accessing the building, but also relates to access to equipment, such as examination tables [8]. It is important to be also aware of geographical and transport related barriers to care, psychological barriers and affordability of care, in particular when people with disabilities also have financial problems due to lack of income compensation schemes or social benefits [31]. More recent conceptualisations look at the interaction of characteristics of the health care services and the people needing the services [32]. Moreover, the focus was on one group of disabilities, namely those that affect people’s ability to walk. Physical access is particularly important for people with mobility restrictions. There are, however, other disabilities, such as mental, visual and hearing disabilities, that bring other access barriers than purely physical accessibility.

### Strengths and limitations

A strong feature of our analysis is that we used a large international dataset on primary care. The data were analysed with state-of-the-art statistical methods that allow for splitting that variation in a part that is related to the countries and a part that is related to the GP practices within the countries.

Another strength is that the assessment was done by relative outsiders (the fieldworkers who visited the practices to interview patients). Mudrick et al. also used data collected by outside reviewers that visited practices for monitoring purposes for health plans; this monitoring included a wider set of criteria because accessibility for people with disabilities was the focus of the assessment [11]. They suggest that outside observers provide more reliable assessments than provider surveys. The actual experiences of people with disabilities in accessing the GP practices were not available.

A limitation is that it was difficult to find relevant information about accessibility of buildings and services in general for people with disabilities for broad range of countries in a comparable and quantifiable way. We used three variables, each of which can be seen as a proxy for what we wanted to measure. A further limitation is that the QUALICOPC data are by now 9 years old. Hence, the relevance of descriptive information is decreasing. However, there are several areas which remain interesting even though the data are a bit older now and the physical accessibility of GP practices is one of these areas. Physical accessibility of buildings probably does not change fast. Moreover, there is a clear lack of country comparative information. We did not have information on the age of the practice buildings and used age of the GPs as proxy for this. This is based on an assumption that we could not test. The QUALICOPC study was not designed for the specific analysis of accessibility. The measurement of accessibility is therefore restricted to only few aspects and only one dimension. In a purpose designed study, further aspects and dimensions should be taken into account.

Our study was conducted mainly in European countries and mostly among member states of the European Union. In this analysis, we used data from two countries outside Europe. Most of the countries participating were high income countries. This limits the generalisability of the results. We do not know whether the associations found, will also be valid in low and middle income countries and outside of Europe.

The large differences between countries implicate that national policies can be used to increase physical accessibility of GP practices. Our analysis also shows that an update of the information on physical accessibility of primary care facilities may be less difficult than one would expect before. The high clustering of accessibility with countries, implicates that a high reliability of the accessibility scale at country level can be reached with a much smaller number of practices per country. Already with 15 practices per country the reliability of the physical access scale would reach 0.90. This means that for monitoring purposes, it is feasible to measure physical accessibility by visiting a relatively small number of practices per country.

## Conclusion

Accessibility of primary care facilities is of prime importance to people with mobility handicaps. Accessibility was assessed by field workers who visited GP practices as part of the data collection for the QUALICOPC study. We found large differences between countries and a strong clustering of physical accessibility within countries. It seems that older premises, single-handed practices and those in in inner city areas are less accessible. Social democratic government participation during the previous decades was positively related to physical accessibility. The fact that a large share of the variation in physical accessibility was on the level of countries, means that national policies can be used to increase physical accessibility of GP practices.

## Supplementary Information

**Additional file 1: Supplementary Table 1.** Background information to the ‘Accessibility regulation index’.

## Data Availability

The datasets used and/or analysed during the current study are available from the corresponding author on reasonable request.

## Abbreviations

FYR Macedonia: Former Yugoslav Republic of Macedonia
GDP: Gross Domestic Product
GP(s): general practitioner(s)
ICC: intraclass correlation
PPP: Purchasing Power Parities
QUALICOPC: Quality and Costs of Primary Care in Europe
UN: United Nations
US(A): United States (of America)
UK: United Kingdom
