# Supplementary material for: Physical accessibility of primary care facilities for people with disabilities: a cross-sectional survey in 31 countries
Source: BMC Health Serv Res. 2021 Feb 1;21:107. doi: 10.1186/s12913-021-06120-0 (PMC7849086; doi:10.1186/s12913-021-06120-0)
Supplement: Supplementary file 1 — Additional file 1: Supplementary Table 1. Background information to the ‘Accessibility regulation index’. [file 12913_2021_6120_MOESM1_ESM.docx]

Supplementary table 1: Background information to the ‘Accessibility regulation index’

| **Country** | **Description in Human Rights Report** | **Law/regulation on accessibility of buildings** | **Restricted to public buildings** | **Problematic implementation/ enforcement** | **Accessibility regulation index** |
| --- | --- | --- | --- | --- | --- |
| **Australia** | The disability discrimination commissioner, who is part of the HRC, promotes compliance with federal laws that prohibit discrimination against persons with disabilities. The commissioner also promotes implementation and enforcement of state laws that require equal access to buildings and otherwise protect the rights of persons with disabilities, including ensuring equal access to communications and information. | **yes** | **no** | **no** | **3** |
| **Austria** | Federal law mandates access to public buildings for persons with physical disabilities; however, NGOs complained that many public buildings lacked such access due to insufficient enforcement of the law and low penalties for noncompliance. Persons with disabilities generally had access to information and communications. They generally were able to vote and participate in civil affairs. | **yes** | **yes** | **yes** | **1** |
| **Belgium** | While the government mandated that public buildings erected after 1970 must be accessible to such persons, many older buildings were still inaccessible. | **yes** | **yes** | **Older buildings not accessible** | **1** |
| **Bulgaria** | The law requires improved access to buildings for persons with disabilities, and most new public works projects took this requirement into account. However, enforcement of this law lagged in existing, unrenovated buildings. | **yes** | **no** | **Older buildings not accessible** | **2** |
| **Canada** | Although the government effectively implemented existing laws and programs mandating access to buildings, information, and communications for persons with disabilities, the absence of uniform standards across the country created disparities in standards of access between provinces. | **yes** | **no** | **No standards** | **2** |
| **Cyprus** | While the law mandates universal accessibility for public buildings and tourist facilities built after 1999, government enforcement was ineffective. Older buildings frequently lacked access for persons with disabilities. | **yes** | **yes** | **Older buildings not accessible** | **1** |
| **Czech Republic** | The government continued its program to aid persons with disabilities, Mobility for All, which focuses on removing access barriers to public buildings in towns and villages, as well as increasing the accessibility of public transportation.  Of Prague’s 54 subway stations, 32 were accessible to persons with disabilities, including five of the 15 major stations in the city center. A majority of buses and new tramcars had low-floor entry doors to accommodate passengers with disabilities. The subway, bus, and tram systems provided stop announcements and equipment for visually impaired riders. | **yes** | **yes** |  | **2** |
| **Estonia** | The law does not mandate access to buildings for persons with disabilities; few older buildings were accessible, although new or renovated buildings generally were.  ...the state provides access to information services and makes individual assistants available to persons with disabilities when necessary. | **no** |  |  | **0** |
| **Finland** | Laws mandating access to buildings for persons with disabilities generally were enforced, although many older buildings remained inaccessible. Most forms of public transportation were accessible, but problems continued in some geographically isolated areas | **yes** | **no** | **Older buildings not accessible** | **2** |
| **FYR Macedonia** | The law requires that only new buildings be made accessible to persons with disabilities. Many public buildings remained inaccessible. Inconsistent inspection resulted in construction of new facilities that were not accessible. NGOs reported the situation was improving over time. Public transportation is largely inaccessible for physically disabled persons. | **yes** | **yes** | **yes** | **1** |
| **Germany** | The federal government continued to provide payments of 518.2 million euros ($673.7 million) per year to the states to support barrier-free buildings, a project scheduled to continue through 2019. Efforts continued to improve barrier-free access to public transportation.  By law the federal government is required to provide barrier-free access to communications, especially in the field of administrative Internet sites and official forms and notifications. | **yes** | **no** | **no** | **3** |
| **Greece** | The law mandates access to buildings for people with disabilities and special ramps for the sidewalks and means of public transportation; however, authorities enforced this law poorly. Activists for the rights of individuals with disabilities reported that parked vehicles often occupied sidewalks and special ramps, thus hindering access for persons with disabilities. The general lack of accessibility forced such individuals to remain at home and led to serious social exclusion. Research by the Polytechnic School of Athens during the year revealed that 69 percent of the country’s ports were not accessible to passengers with disabilities. The law allows service animals to accompany blind people in all mass transit, but blind activists claimed they faced serious problems when attempting to use city transportation with their dogs.  Only 5 percent of public buildings were fully accessible, with the majority of these in Athens; for the most part, even buildings with special ramps did not have accessible elevators or lavatories. The deputy ombudsman for social welfare handled complaints related to people with disabilities, especially those related to employment, social security, and transportation. | **yes** | **no** | **yes** | **2** |
| **Hungary** | Both the central government and municipalities continued to renovate public buildings to make them accessible to persons with disabilities. The law originally set 2010 as the deadline by which the central government had to make buildings accessible, while municipalities have until 2013. There was no data available on the percentage of government buildings that complied with the law, but NGOs contended many public buildings remained inaccessible. | **yes** | **yes** | **Yes (according to NGOs)** | **1** |
| **Iceland** | The government ensured that persons with disabilities had access to buildings, information, and communications. Disability rights advocates complained that access to public information was dissatisfactory, since not all persons with disabilities had electronic access. Building regulations require that public accommodations and government buildings, including elevators, be accessible to persons in wheelchairs; that public property managers reserve a minimum of one space for persons with disabilities; and that sidewalks outside the main entrance of such buildings be kept clear of ice and snow to the extent possible. While violations of these regulations are punishable by a fine or a jail sentence of up to two years, the main association for persons with disabilities complained that authorities rarely, if ever, assessed penalties for noncompliance. | **yes** | **yes** | **yes** | **1** |
| **Ireland** | The government effectively implemented laws and programs to ensure that persons with disabilities have access to buildings, information, and communications. The National Disability Authority has responsibility for setting and implementing disability standards, as well as directing disability policy. | **yes** | **no** | **no** | **3** |
| **Italy** | Although the law mandates access to government buildings for persons with disabilities, mechanical barriers, particularly in public transport, continued to pose challenges. Many cities lacked infrastructure (such as subway elevators, funicular stations, and ramps on sidewalks) for persons in wheelchairs or with limited mobility. | **yes** | **yes** | **yes** | **1** |
| **Latvia** | The law mandates access to air travel and other transportation, and during the year the government and municipalities had partially implemented the law. The also law mandates access to buildings for persons with disabilities; however, most buildings were not accessible. | **yes** | **no** | **yes** | **2** |
| **Lithuania** | The law mandates accessibility of buildings for persons with disabilities. According to 2011 data from the Department of Statistics, nearly 45 percent of housing was accessible to persons with disabilities.  The Ministry of Health is responsible for making health services equally accessible to all inhabitants of the country. | **yes** | **No, healthcare explicitly mentioned** | **no** | **3** |
| **Luxembourg** | The law does not require government or privately owned buildings to be accessible to persons with disabilities, but the government subsidized the construction of such structures. | **No law, bus subsidies available** | **-** |  | **3** |
| **Malta** | The government previously implemented legislation to ensure access to buildings, information, and communication. In November the government launched a Maltese language voice recognition software for use by persons with speech and hearing disabilities. | **yes** | **no** | **?** | **3** |
| **Netherlands** | The law also requires that persons with disabilities have access to public buildings, information, and communications, but, despite continued progress, public buildings and public transport were not always easily accessible in practice. | **yes** | **yes** | **yes** | **1** |
| **Norway** | The law applies to all persons with disabilities without enumerating specific types of disabilities. It mandates access to public buildings, information, and communications for persons with disabilities. | **yes** | **yes** |  | **2** |
| **Poland** | The law states that buildings should be accessible for persons with disabilities, and at least three laws require retrofitting of existing buildings to provide accessibility. Many buildings remained inaccessible to persons with disabilities, however, because regulations do not specify what constitutes an accessible building. Public buildings and transportation generally were accessible although older trains and vehicles are often less accessible to persons with disabilities, and many train stations are not fully accessible for all types of disabilities. | **yes** | **no** | **yes** | **2** |
| **Portugal** | The law also mandates access to public buildings for persons with disabilities, and the government implemented these provisions in practice; however, no such legislation covers private businesses or other facilities. | **yes** | **yes** | **no** | **2** |
| **Romania** | The law mandates accessibility for persons with disabilities to buildings and public transportation. In practice the country had few facilities specifically designed to accommodate persons with disabilities, and persons with disabilities could have extreme difficulty navigating city streets or gaining access to public buildings. However, the number of buildings with facilities for persons with disabilities increased during the year. In addition, the National Library was the first public institution to receive a disability access certificate in December. | **yes** | **no** | **yes** | **2** |
| **Slovakia** | In practice, however, experts reported that access to buildings and higher education remained a problem  While the law defines mandatory standards for access to buildings, NGOs noted they were not fully implemented, although access to privately owned buildings improved more rapidly than access to state buildings. | **yes** | **no** | **yes** | **2** |
| **Slovenia** | The law mandates access to buildings for persons with disabilities, but modification of public and private structures to improve access continued at a slow pace, and many buildings were not accessible in practice. The government continued to implement laws and programs to provide persons with disabilities with access to buildings, information, and communications. | **yes** | **yes** | **yes** | **1** |
| **Spain** | The law mandates access to buildings for persons with disabilities, and the government generally enforced these provisions; however, levels of assistance and accessibility differed between regions. | **yes** | **no** |  | **3** |
| **Sweden** | Regulations for new buildings require full accessibility, and similar requirements exist for some, but not all, public facilities. However, many buildings and some means of public transportation remained inaccessible. | **yes** | **no** | **Yes** | **2** |
| **Switzerland** | The law mandates access to public buildings and government services for persons with disabilities, and the government generally enforced these provisions in practice. | **yes** | **yes** | **No** | **2** |
| **Turkey** | The law requires all governmental institutions and businesses to make necessary arrangements for access for persons with disabilities in public areas and on public transportation by July 2011, but the government made little progress implementing the law. Access in most cities was extremely limited, and there was no clear system of fines or other punishment for noncompliance. | **yes** | **no** | **Yes** | **2** |
| **UK (England)** | The law requires that all public service providers (except in the transportation sector) make “reasonable adjustments” to ensure their services are available to persons with disabilities | **yes** | **yes** |  | **2** |
